# The Story of the Insane from Year to Year

**Published:** 1895-07-06

**Authors:** 


### THE STORY OF THE INSANE FROM YEAR TO YEAR.

### HEREFORD COUNTY AND CITY ASYLUM.

Here the numbers went down from 394 to 386. There were 95 admissions, 29 recoveries, 31 discharged relieved, and 22 deaths. The percentage of recoveries calculated on the admissions was 30·5, and that of the deaths calculated on the average number resident was 5·6. Nine of the deaths were due to brain diseases, and 4 to consumption. Intemperance in drink caused the insanity in 7 of the admissions, domestic trouble in 9, hereditary influence in 24, and previous attacks in 14. The asylum is full, and patients are boarded out, of course at an increased cost to the ratepayers, in the Gloucester Asylum and at Grove Hall, Bow, there being 10 at the former asylum and 20 at the latter. It is not likely that the payments for these are less than 14s. a week per head, and as the weekly cost at Hereford is a fraction over 9s., we have a loss to the county ratepayers of nearly £400 a year, besides the pain and inconvenience which must result to patients from having their relatives sent from home. In large asylums a committee may be forgiven if they think twice before building additions, but at Hereford there need be no hesitation. A large sum of money is being paid every year, and these payments do not prevent building operations —they merely delay them for a few years. In the meantime Dr. Chapman is doing what he can to obtain space. Twenty-one chronic cases have been discharged to workhouses, and we are not surprised to learn that six have already been returned to the asylum. The Committee of Visitors "speak with the same confidence and satisfaction of Dr. Chapman's able superintendence as in former years."

### CONNECTICUT HOSPITAL FOR THE INSANE.

This is a large asylum, containing at the beginning of the year 1,535 patients, and at the close of the year the number had risen to 1,580. The admissions were 358, the recoveries 63, discharged relieved 68, and the deaths 101. Dr. Olmstead says that "barely one-third of 358 persons admitted last year were in such a condition as would justify expectation of recovery even in the most favourable environment," so that it would seem as if the asylums of the New World had as many chronic cases sent to them as those of the Old World. Dr. Olmstead's report shows that he by no means neglects the purely medical part of the treatment of the insane; but he evidently sets great store on the employment of patients, and when speaking of "escapes," we have him saying that the benefit derived from occupation overbalances occasional escapes. This "occupation or industrial treatment, as someone has put it, and aptly, too, for the things that may engage an insane person's attention, and may keep his hands out of mischief, are by no means beneath the notice of the physicians, and when among several hundred cases of chronic insanity not a dozen are ailing, but many idle, surely it is strictly within the bounds of medical work to devise various methods of keeping as many as possible busy in healthful ways." The weekly cost of maintenance is about 11s. 6d. per head.

